# Supplementary material for: The relationship between workload and burnout among nurses: The buffering role of personal, social and organisational resources
Source: PLoS One. 2021 Jan 22;16(1):e0245798. doi: 10.1371/journal.pone.0245798 (PMC7822247; doi:10.1371/journal.pone.0245798)
Supplement: S1 Table — (DOCX) [file pone.0245798.s001.docx]

S1 Table. Number of questionnaires sent out to facilites and response rate

| Questionnaires sent out | Outpatient care | | | Hospitals | | | Nursing homes | | | *Not attributable** | In total | | |
| --- | --- | --- | --- | --- | --- | --- | --- | --- | --- | --- | --- | --- | --- |
|  | Sent out | Response | **Response rate (%)** | Sent out | Response | **Response rate (%)** | Sent out | Response | **Response rate (%)** | Response | Sent out | Response | **Response rate (%)** |
| Paper | 327 | 80 | 24,5 | 160 | 29 | 18,1 | 1777 | 315 | 17,7 | *21* | 2264 | 445 | **19,7%** |
| Online | 329 | 16 | 4,9 | 0 |  |  | 389 | 31 | 8,0 | *5* | 718 | 52 | **7,2%** |
| **In total** | **656** | **96** | **14,6** | **160** | **29** | **18,1** | **2166** | **346** | **16,0** | ***26*** | **2982** | **497** | **16,7%** |

*By contacting and sending out the questionnaires these facilities were either outpatient care facilities, hospitals or nursing homes. This item was missing on these completed questionnaires.
